# E Before A: Awake Bi-femoral Veno-Venous Extracorporeal Membrane Oxygenation as a Bridge to the Bifurcation Tracheal Y Stent

**DOI:** 10.7759/cureus.76890

**Published:** 2025-01-04

**Authors:** Elliott T Worku, Michael G Pittard, Ruaidhri Carey

**Affiliations:** 1 School of Medicine, University of Sydney, Sydney, AUS; 2 Intensive Care Unit, Royal Prince Alfred Hospital, Sydney, AUS

**Keywords:** airway procedures, awake technique, central airway obstruction, hypoxic respiratory failure, non-small cell lung carcinoma (nsclc), tracheal stenting, veno-venous extracorporeal membrane oxygenation (vv ecmo)

## Abstract

A 64-year-old female presented with severe respiratory failure secondary to a high-grade non-small lung cancer (IIIA NSCLC) causing extrinsic and intrinsic compression of the right main bronchus. She remained hypoxic despite 100% FiO_2_ delivery by high-flow nasal cannula and was considered at high risk of airway loss at intubation. Tumor debulking, histological diagnosis, and restoration of airway patency were facilitated with peri-procedural veno-venous extracorporeal membrane oxygen support (VVECMO).

We describe a case of awake bifemoral VVECMO cannulation performed uneventfully as a bridge to the palliative placement of a self-expanding tracheal Y stent. The circuit was maintained in the absence of systemic anticoagulation. After less than 24 hours of extracorporeal support, the patient was decannulated, liberated from supplementary oxygen, and discharged from intensive care. The patient is now receiving platinum-based chemoradiotherapy and is eligible for targeted consolidative immunotherapy.

As therapies and practice evolve, extracorporeal support may serve as a bridge to palliative interventions intended to salvage and improve the quality of life in oncology patients. Awake cannulation is feasible and may be preferred in cases of malignant airway obstruction.

## Introduction

Hypoxic respiratory failure resulting from malignant central airway obstruction portends extremely high mortality. Airway instrumentation, while critical to relieving hypoxia, restoring airway patency, and obtaining a histological diagnosis, risks irrevocable airway collapse. To provide a safe environment to intubate the trachea and perform rigid bronchoscopy, veno-venous extracorporeal membrane oxygenation (VVECMO) may solely support gas exchange in the absence of native lung ventilation.

VVECMO comprises a large cannula providing venous drainage and venous return, a centrifugal pump, and a membrane oxygenator. The therapy drains blood typically from the inferior vena cava, right atrium, or superior vena cava, and after exogenous addition of oxygen and carbon dioxide removal, returns this *arterialized* blood to the right atrium. This post-membrane blood often demonstrates supraphysiological PO2 (>400 mmHg), and there is an admixture between the blood exposed to the ECMO circuit and the residual fraction of cardiac output, which passes through the patient's native lungs without extracorporeal support. This invasive and costly therapy is typically provided for the archetypal indication of acute respiratory distress syndrome (ARDS), in severely hypoxemic patients, to support oxygen delivery and permit reductions in mechanical ventilation intensity to limit injury. It can be used to bridge patients toward lung transplantation or recovery, and thus palliative diagnoses, frailty, or other co-morbidities of significance generally preclude candidacy for ECMO. Complications are common, including consequences of altered coagulation (bleeding, thrombosis, and intracranial hemorrhage), and sequelae of cannulation such as infection and vascular injury. Periprocedural use of ECMO and indeed awake implantation of VVECMO are increasingly reported.

There may be utility as a compassionate bridge to palliative relief of airway obstruction. Judicious use of ECMO to facilitate tracheal stent implantation, perform biopsy to inform prognosis and therapeutic strategy, and provide symptomatic relief may avoid futility and be of benefit to patients with unresectable locally advanced malignancy.

We present a successful case of awake femoral cannulation in a patient with central airway obstruction, permitting safe induction of anesthesia, cryobiopsy, and stenting of the tracheobronchial tree with a self-expanding Y stent. Periprocedural use of ECMO outside of traditional indications is expanding, and experienced centers may achieve excellent outcomes in previously underserved patient cohorts.

## Case presentation

**Figure 1:**
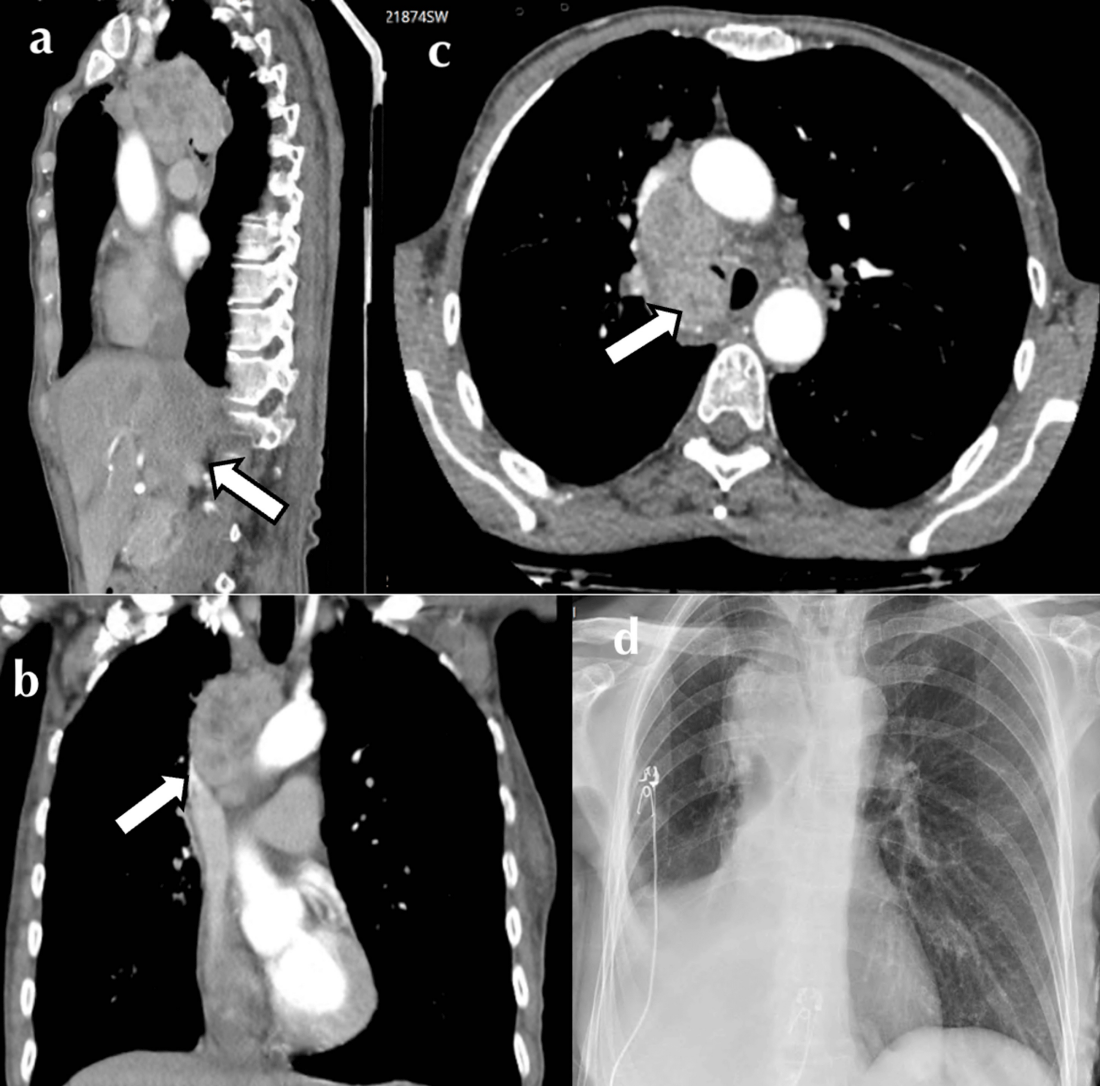
Computed tomography (CT) and chest X-ray (CXR) imaging demonstrating a large mixed-density mediastinal mass causing right main bronchus compression and right middle and lower lobe collapse: (a) sagittal, (b) coronal, (c) axial, and (d) AP-CXR.

A 64-year-old female was referred to our tertiary service with malignant central airway obstruction. She was a 60-pack-year smoker and treated hypertensive with a two-month history of cough, hemoptysis, anorexia, and 10 kg unintentional weight loss. Positron emission scan (PET-CT) imaging revealed an irregular 28 mm x 26 mm x 19 mm right upper lobe mass with central necrosis (Figure 1). There was associated ipsilateral hilar and paratracheal FDG-avid lymphadenopathy encircling the right main bronchus, with a mass effect on the SVC also noted. Her disease (IIIA) was deemed inoperable; histology was required to further differentiate between small and non-small cell neoplasm and elicit candidacy for immunotherapy.

Initial plans for intervention were foiled by rapidly progressive and severe hypoxic respiratory failure, with PaO_2_:FiO_2_ ratio <100 mmHg (range 65-87 mmHg) consistent with severe acute hypoxemic respiratory failure despite maximal supplemental high-flow nasal oxygen. Intubation posed a risk of central airway collapse, and the patient's habitus was incompatible with dual-lumen endotracheal intubation, which also would not have provided adequate procedural access. Given the luminal intrusion of the mass, debulking was required with the potential for airway hemorrhage contamination of the aerated lung and further luminal compromise. Jet ventilation in conjunction with suspension laryngoscopy to facilitate stenting was considered but was deemed unlikely to sufficiently overcome the degree of hypoxia. Additionally, there was concern regarding the adequacy of passive expiration, as its failure could lead to life-threatening barotrauma [1,2].

Surgery was delayed, and the patient was admitted to the intensive care unit for further supportive management. Chest X-ray demonstrated compression of the right tracheobronchial take-off with the collapse of the right middle and lower lobes (Figure 1). Non-invasive ventilation was trialed but poorly tolerated. Hypoxia was exacerbated in the right lateral position, likely due to an aggravation of V/Q mismatch. As a result, nebulized pulmonary vasodilator therapy was initiated to ameliorate the pulmonary shunt, and empiric antibiotic therapy was also administered due to post-obstructive consolidation. A multidisciplinary meeting was held between the Intensive care, thoracic medicine, and ECMO specialists. Periprocedural VVECMO was offered as a bridge to rigid bronchoscopy, tumor debulking, and tracheal stenting, with hopes of rapidly reversing respiratory failure.

Given the presence of intrathoracic extrinsic compression, abolition of spontaneous ventilation was fraught; thus, awake bifemoral ECMO cannulation was planned.

The patient was administered sub-dissociative aliquots of intravenous ketamine, and a continuous dexmedetomidine infusion was commenced at 0.6 mcg/kg/minute. Local anesthesia was infiltrated into the femoral sites, after which 6 Fr and 5 Fr vascular sheaths were placed in the right and left common femoral veins, respectively, by ultrasound-guided vessel puncture. Under subcostal transthoracic echo guidance, two 180 cm Amplatz super stiff wires (Boston Scientific, Boston, MA) were advanced until visualized at the cavo-atrial junction. A bolus of intravenous heparin (2,500 IU) was administered, and following sequential percutaneous tract dilation, a short 25-Fr, 38-cm multistage access cannula was advanced to the cavo-atrial junction, followed by a 21-Fr, 55-cm single-stage return cannula to the right atrium. A HLS 7.0 Bioline® circuit (Maquet GmbH, Getinge, Rastatt, Germany) was connected, and V_cfvr ivc _25/38 - V_cfvl ar _21/55 ECMO was initiated at 3 LPM extracorporeal blood flow, 2 LPM sweep gas flow (SGFR), and an oxygenator blend fraction (FbO2) of 1.0.

**Figure 2:**
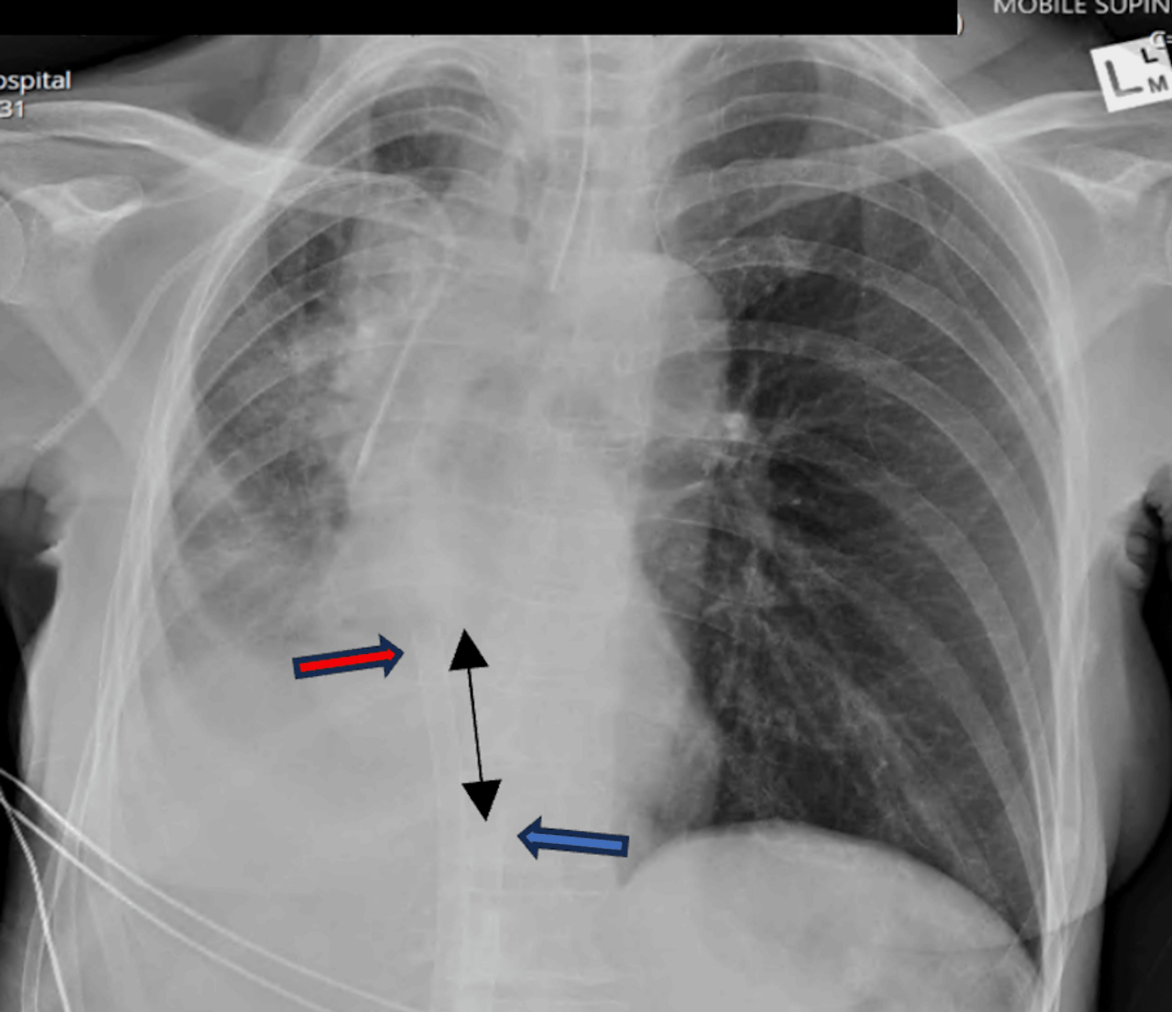
CXR post VVECMO cannulation. Multistage access cannula at the cavo-atrial junction (blue arrow) and single-stage return cannula (red arrow) positioned in the mid-right atrium. CXR, chest X-ray; VVECMO, veno-venous extracorporeal membrane oxygen

The Cardiohelp console (Maquet GmbH, Getinge, Rastatt) displayed an SvO2 of 65%, with an appropriate color differential between access and return excluding significant recirculation. Patient oxygen saturation rapidly improved to 100%. Following uneventful anesthetic induction, endotracheal intubation with a single-lumen endotracheal tube was performed. Post-intubation transoesophageal echo and CXR confirmed appropriate positioning of the ECMO cannula (Figure 2).

**Figure 3:**
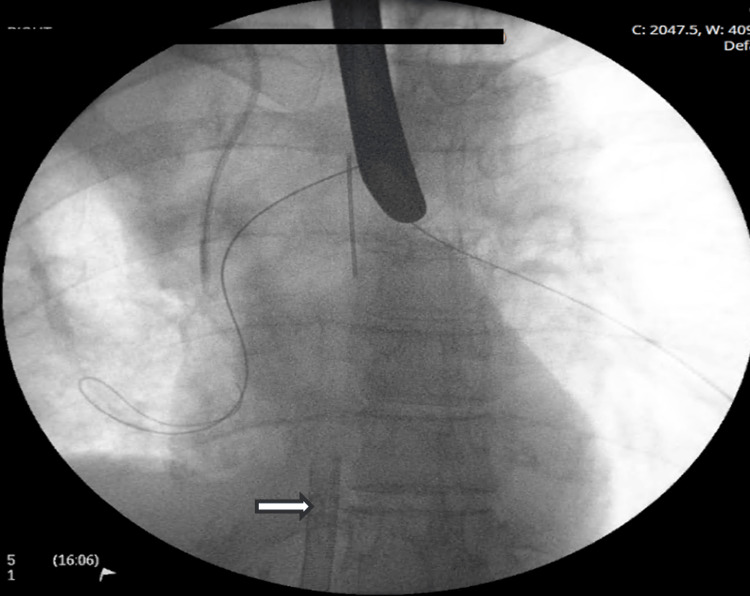
Fluoroscopy: Rigid bronchoscopy deployment of bifurcation wires to guide tracheal Y stent during apnoeic period. The VVECMO single-stage return cannula is denoted by the white arrow. VVECMO, veno-venous extracorporeal membrane oxygen

The patient promptly underwent ECMO-supported rigid bronchoscopy, tumor debulking, and cryobiopsy (Figure 3). Fluoroscopically guided balloon dilation was performed on the right bronchial tree.

**Figure 4:**
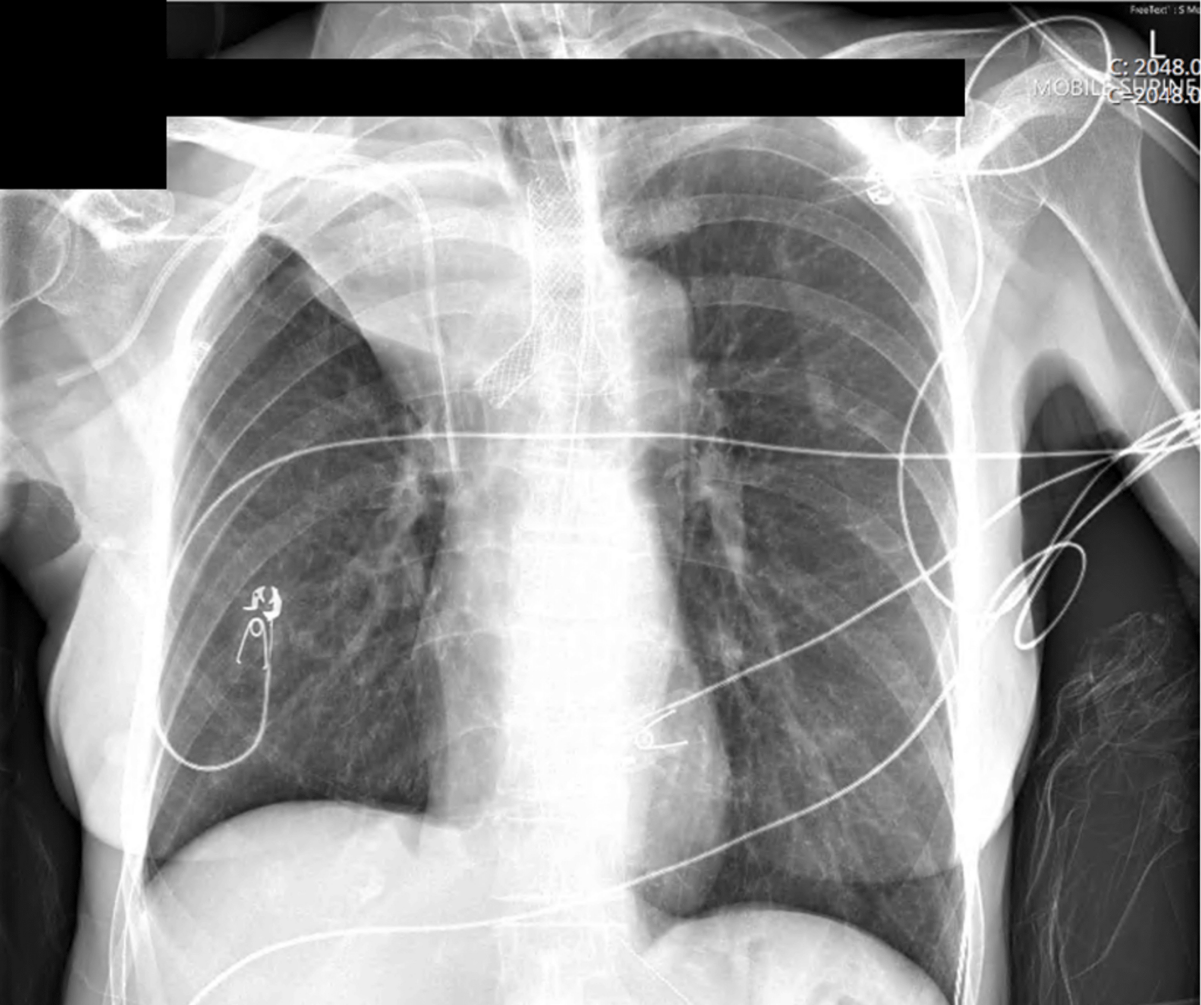
Postoperative AP CXR demonstrating reversed nitinol-coated Y stent (note: position with long left main bronchus projection intentionally deployed to the RMB) with sacrifice of the right upper lobe (RUL), but restoration of right middle lobe (RML) and right lower lobe (RLL) aeration. AP CXR, anteroposterior chest X-ray; RMB, right main bronchus

Thereafter, a Leufen Aerstent® (Leufen Medical GmbH, Berlin) self-expanding, silicone-covered nitinol Y stent was placed. The stent was asymmetrical, and in this case, the longer sidearm, typically reserved for the left main bronchus, was intentionally rotated and placed in the right main bronchus, as this provided sufficient extension beyond the margin of the residual luminal compression (Figure 4).

Postoperative imaging demonstrated re-expansion of the right middle and lower lobes, but anticipated sacrifice of the right upper lobe (Figure 4). The patient returned to ICU, and ECMO was continued overnight without systemic anticoagulation, maintaining nadir flows of 3 LPM to mitigate the risk of thrombosis, while both SGFR and blend were iteratively weaned (nadir 1 LPM SGFR and FbO2 21%).

The following morning, a brief sweep gas-off trial was conducted, after which the patient was decannulated in the ICU with purse-string sutures and extubated to room air. As per local protocol, lower limb Doppler sonography was performed after the removal of the ECMO cannula, which revealed a small, non-occlusive left common femoral vein thrombus. This was asymptomatic and may have been cannula-related, or preexisting in association with immobility and malignancy. On the balance of her prothrombotic state, therapeutic oral anticoagulation was commenced. Many centers do not routinely surveil for deep vein thrombosis (DVT) post-ECMO; however, we performed this as part of our decannulation package of care and observed ~25% rate of DVT, of which few are symptomatic. Whether the DVT was associated with ECMO cannulation or was a consequence of ECMO remains unclear.

Histology confirmed non-small cell adenocarcinoma and subsequent immunohistochemistry demonstrated TP53 expression. The patient was discharged to undergo six weeks of outpatient treatment, which included 60 Gy of radiotherapy in divided fractions, platinum-based doublet chemotherapy (Carboplatin and Paclitaxel), and consolidative Durvalumab immunotherapy (with 40% expression of the PD-L1 ligand). At the time of reporting, she had ceased oral anticoagulation, had undergone airway stent retrieval, and was at home with her family.

## Discussion

This case demonstrates periprocedural VVECMO as a salvage technique for obstructive hypoxia and a bridge to palliative restoration of airway patency.

While the mass was known to represent a high-grade malignancy, histological and immunohistochemical characterization was not possible without extracorporeal support, and the patient was likely to die imminently from hypoxic respiratory failure.

Bifurcation Y stents are typically deployed via a rigid bronchoscope and directed over guidewires placed into each main bronchus (Figure 3) [2]. Stent placement typically involves a period of apnea to limit movement and malposition; this would have been poorly tolerated in our patient without ECMO. While other techniques, including suspension laryngoscopy and jet ventilation, have been described, these carry the risk of potentially lethal barotrauma due to obstruction of passive exhalation [1].

In a multicenter experience of 38 stent placements (the majority in malignant airway obstruction), the median procedural duration was 37.6 (29.8.45.4) minutes [2]. This is a protracted period for a hypoxic patient with interruptions to ventilation, and the potential for contralateral lung soiling and airway loss. Despite the lengthy instrumentation, procedural success was achieved in 37/38 patients, with rapid resolution of respiratory failure occurring thereafter. Despite zero procedural mortality and low rates of postoperative complications, 18 patients succumbed to their disease at 12 weeks [2]; thus, airway stenting represents a palliative intervention with significant implications for short-term survival and quality of life.

While we traditionally perform femoral-jugular VVECMO cannulation, a bifemoral approach was chosen for improved tolerance in the awake patient. Femoral instrumentation carries a low risk of air entrainment during spontaneous ventilation, and given profound respiratory embarrassment, Trendelenburg was undesirable. Furthermore, tumor compression of the superior vena cava presented a relative preclusion to jugular return cannulation.

We used a short 38-cm multistage access cannula instead of the conventional 55-cm cannulas, chosen due to the patient's habitus and because implantation was performed under transthoracic echocardiographic guidance, which cannot provide a bi-caval view to facilitate safe passage to the SVC. While systemic anticoagulation is usually indicated during ECMO support, this can be omitted to broaden the application of ECMO to situations when bleeding risk is elevated [3], and the consequences would be catastrophic. 

VVECMO was undertaken explicitly as a bridge to induction, restoration of airway patency, and diagnosis. The patient and family were counseled that prolonged postoperative ECMO would not be appropriate, nor would escalation to mechanically circulatory support be provided in case of major hemodynamic decompensation or circulatory arrest. Veno-arterial ECMO has been successfully employed to manage airway obstruction due to malignant thyroid cancers; however, in our case, it would have carried a substantial risk of differential hypoxia [4].

Short-term ECMO support of oncology patients is expanding to facilitate relief of malignant airway obstruction [4,5], chemotherapy induction in thoracic cancers [6], and modified extracorporeal circuits have been used to deliver isolated lung perfusion to increase local chemotherapeutic potency while sparing systemic toxicity [7].

The Extracorporeal Life support registry cites immunosuppression as a relative contraindication to ECMO, but “anticipated non-recovery without a plan for viable decannulation” as the only absolute contraindication [8]. VVECMO remains a bridging therapy, and while it is usually used for recovery or transplantation, short-term use to ameliorate perioperative risks and achieve palliative improvements in quality of life may not be antithetical to this.

## Conclusions

We describe one example of VVECMO outside of traditional indications, best offered by experienced centers with multidisciplinary consultation. Prior planning is important to minimize morbidity from ECMO and ensure the benefit is realized in high-risk cohorts. It is crucial to preemptively define appropriate escalations in case of antecedent or periprocedural patient deterioration.

Awake cannulation to VVECMO may be useful in patients for whom a physiologically challenging airway is predicted, but it requires careful patient management and familiarity with alternative imaging guidance. The significant risks of ECMO must be carefully mitigated to avoid non-beneficence in these high-risk cases. Anticoagulation-free ECMO is feasible where procedural risks of bleeding are anticipated, but risks of thrombosis may be increased. In high-volume centers with established ECMO services, expanding periprocedural support may be considered.

Judicious use of ECMO may dramatically improve short-term outcomes in patients at risk of terminal decompensation from malignant central airway obstruction. Short-term support with pre-defined limitations may help avoid a *bridge-to-nowhere* scenario.
